# Rheo-Impedance Behavior during the Gelation Process of a Gelatin Solution

**DOI:** 10.1021/acsomega.5c02177

**Published:** 2025-06-09

**Authors:** Yoshifumi Yamagata, Keisuke Miyamoto

**Affiliations:** † Anton Paar Japan K. K., Riverside Sumida 1Fl, 1-19-9, Tsutsumi-dori, Sumida-ku, Tokyo 131-0034, Japan; ‡ Faculty of Science and Technology, Tokyo University of Science, 2641 Yamazaki, Noda-shi, Chiba 278-8510, Japan

## Abstract

Since a gelatin solution easily undergoes a reversible sol–gel phase transition by heating and cooling, it has been the subject of research using various approaches for a long time. However, there have been few studies that have dynamically and in real time evaluated the series of processes that transform a sol to a gel (or from a gel to a sol) via the sol–gel transition. We therefore decided to use the rheological impedance technique, which can be measured in real time, to evaluate the dynamic changes in the internal structure of the gelatin during the transition process from the sol to the gel during cooling. The point at which the elastic moduli (*G*′ and *G*″) of the gelatin rapidly increased shifted to a lower temperature and time with the increasing cooling rate. The *G*′ at the point where the gelation converged was higher for the slower cooling rates. The impedance |*Z*| during the gelation process increased with decreasing temperature. The resistance value, *R*_1_, which is thought to be due to the internal structure of the gelatin, exponentially increases until near the sol–gel transition. Thus, it seems that *R*_1_ indicates the resistance of electrolytes and ions moving inside the cross-linked collagen molecules. Since the apparent plateau modulus *G*′_p_ is in a proportional relationship with *R*_1_, we concluded that *R*_1_ corresponds to the increase of molecular weight accompanying the progress of the cross-linking. Therefore, we found that the rheo-impedance measurement is a very effective method for examining the dynamic and real-time changes in the internal structure of the gelatin during the entire sol–gel transition process.

## Graphical abstract

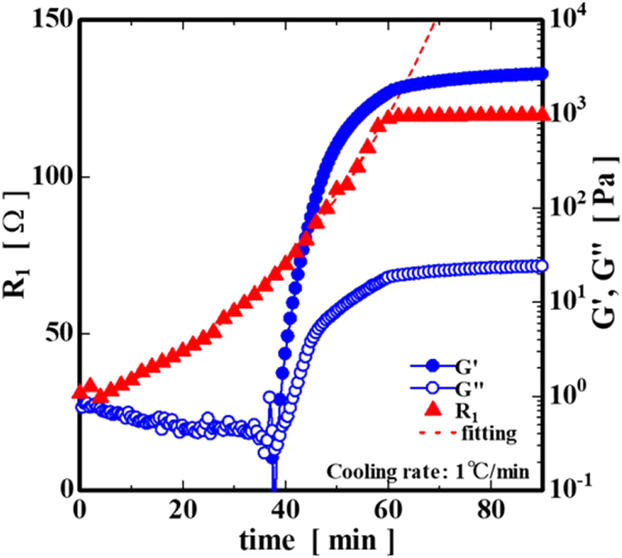

## 1 Introduction

It is said that gelatin’s origins date back to ancient Egypt, where it was used as an adhesive for wooden objects buried in tombs. Nowadays, gelatin is used in a wide range of fields, including not only as an adhesive but also in the production of Japanese and Western sweets, as a binding agent for photos, X-ray film, and photographic paper, and as a coating agent for capsules and tablets in the medical field. Its usefulness is significant, and it is expected to be applied in many more fields in the future.^1–4^

The gelatin solution is normally a sol fluid above 40 °C and maintains a flexible random coil structure. The gelatin molecules partially self-aggregate into a helix structure during cooling. Furthermore, if the concentration is sufficient, a three-dimensional network structure is formed, and it loses its fluidity and becomes a gel. This process is said to be almost identical to percolation theory.^5^

The gelled gelatin easily reverts to the sol with heating, and a reversible sol–gel phase transition occurs. The phase transition process caused by heating and cooling is interesting not only from the perspective of culinary science but also from the physical chemistry of gels, so rheological research has been conducted for a long time.^6–12^ According to past research reports, the viscoelastic behavior of the gelatin during the cooling process is such that the higher the concentration or molecular weight of the gelatin, the higher the increase in the storage modulus *G*′ accompanying gelation will be on the high-temperature, short-time side, and the *G*′ value at the point where gelation has converged will also tend to be high.^13–15^ In addition, since gelatin is an amphiphilic material, it is sensitive to the pH of the surrounding environment.^15–17^ For example, if you prepare a gelatin solution with pH = 2 or 12, we will get a soft gel. On the other hand, if we prepare it in the range of pH = 5–10, which is close to the isoelectric point, we will get a harder gel. Under high acidity and high alkalinity, the gelatin surface acquires a positive and negative charge, respectively, which inhibits the formation of a dense helical structure and reduces the physical cross-linking network density. In other words, this means that the elastic modulus of the system depends on the amount of the helical structure, and that the elastic modulus and the amount of the helical structure can be expressed by a single curve, regardless of the thermal history.^16,18^

Furthermore, the viscoelastic behavior of gelatin is also affected by the cooling rate. Kawamura et al.^19^ evaluated the gelation behavior during the cooling in two ways: refrigeration (slow cooling) and ice water storage (rapid cooling), with an awareness of the cooking plan. They reported that the slow cooling in a refrigerator takes longer for the sample to gel, and the gelation temperature is also significantly higher than the rapid cooling in ice water. Fonkwe et al.^20^ also evaluated the relationship between the cooling rate and the time and temperature of the gelation point, and reported that no gel forms when the cooling rate is faster than 4 °C/min, but that when the cooling rate is slower, the time of the gelation point becomes longer and the temperature shifts to a higher value. This is possibly due to the slower cooling rate, and a more advanced ordered (helical) structure can be formed. In other words, the amount of the helical structure contained in the gelatin solution is a major factor affecting the gelation, and it can be said that the elastic modulus appears when the helical structure is connected in three dimensions.

As already mentioned, the gelation behavior of the gelatin has been investigated from the perspective of its viscoelastic properties by many researchers, but all of these studies targeted the state in which the three-dimensional cross-linking of the collagen has infinitely developed and the elasticity has appeared. The cross-linking of collagen molecules is gradually progressing even in the sol, and via the sol–gel transition process, it eventually spreads to infinity and transitions to the gel; however, the change from the sol to the sol–gel transition is difficult to evaluate using the viscoelasticity technique.

On the other hand, the behavior from the sol to near the sol–gel transition has been studied by the optical rotation technique.^6,21^ The gelatin that is a sol at high temperatures maintains a flexible random coil structure and has a specific optical rotation of −100 to −200°. When cooled, the collagen molecules partially form a helix structure and the optical rotation appears changing the specific optical rotation to −300 to −450°.^22^ According to Kobayashi et al.,^23^ the increasing rate of the specific optical rotation during the initial stage of cooling is faster the higher the specific optical rotation value that is reached, i.e., the higher the concentration of the solution. In addition, the change in the optical rotation shows first- and second-order concentration dependences in the range of a 1–2% gelatin concentration. Furthermore, Tadda^24^ has also reported that the specific rotation (−[α]_D_) is proportional to the square root of the gel strength (−[α]_D_ ∝ *G* ^1/2^). As already mentioned, the changes in the optical rotation are postulated to be a useful indicator for tracking the gelation process, but because the optical rotation measurement is static, it is difficult to evaluate the dynamic changes.

Therefore, we focused on the rheo-impedance measurement, in real time, which can measure both the rheological and electrochemical properties. This method involves measuring the viscosity or the elastic moduli (storage modulus and loss modulus) while applying a dynamic shear flow or strain and simultaneously measuring the impedance of the sample. It has recently been used in various industrial fields. For example, Shitanda et al.^25^ evaluated the rheo-impedance technique to evaluate the dispersibility of acetylene black in an electrode slurry dispersion, and found that samples with good dispersibility had low conductivity when the shear applied. Yamagata et al.^26^ applied these techniques to cosmetics field and evaluated the change in feeling during the use of a commercial O/W cleansing cream. According to them, the changes in the rheological and impedance properties of the cleansing cream when shear is applied correspond to each other, and they conclude that the changes in feel when using it are due to the phase transition from oil-in-water to water-in-oil.

As already described, the rheo-impedance measurement is a technique that can capture the dynamic changes in real time, and it is being used in a variety of fields to obtain a great deal of useful information. We therefore decided to perform the rheo-impedance measurements in order to dynamically and in real time capture the behavior of gelatin from the sol to near the sol–gel transition point or the gel during the cooling process, which had not been fully evaluated until now. Furthermore, we decided to simulate an equivalent circuit model from the impedance obtained in the measurement and considered the changes in the internal structure of the sample during the gelation process from the perspective of the electrical characteristics.

## 2 Methods

### 2.1 Materials and Sample Preparation

The gelatin used for the measurement was bovine bone gelatin (type B, lot 211116, Nitta Gelatin, Inc.), which was used without any pretreatment or purification. The molecular weight of the gelatin, as measured using the PAGI method,^27^ was approximately 170,000.

A 2.5% gelatin dispersion was heated to approximately 65 °C and stirred for 10 min to obtain a uniform gelatin solution. It was then allowed to naturally cool at room temperature and then stored in a refrigerator.

### 2.2 Rheo-Impedance Measurement

Figure 1 shows a schematic diagram of the rheo-impedance measurement device. The impedance measurement geometry (Anton Paar GmbH, Graz, Austria) was attached to the stressed-controlled rheometer (MCR302e, Anton Paar GmbH, Graz, Austria) and connected to the impedance measurement device (IM-3536 LCR meter, HIOKI E. E. Corp., Nagano, Japan) with a BNC/SMB conversion harness. The upper and lower plate jigs for a rheometer are the electrodes.

**Figure 1:**
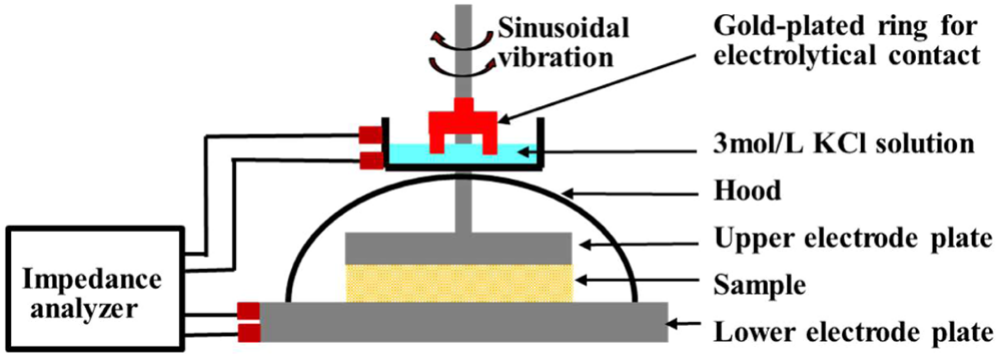
Schematic diagram of rheo-impedance device.

To suppress the sinusoidal vibration noise of the upper plate jig of the 25 mm diameter, a gold-plated clip was attached to the shaft of the upper plate, and conductivity was achieved via a 3 mol/L KCl solution.^25,26^ The gap between the samples was set at 0.5 mm as the distance at which both the viscoelasticity and impedance data could be obtained appropriately.

The sample was melted by heating to approximately 65 °C and then dropped onto the electrode plate, and the dynamic viscoelasticity was measured under the conditions of 1% strain and 10 rad/s angular frequency. The temperature was cooled from 65 to 5 °C at three levels of speed, i.e., 1, 2, and 4 °C/min, then held at 5 °C. In addition, the impedance measurement was also carried out in synchronization with the dynamic viscoelasticity one.

The impedance measurements are conducted by applying an alternating signal (voltage *V*(ω) or current *I*(ω)) to a sample placed between upper and lower electrode plates, and the electrical resistance (impedance) are obtained using eq 1 calculated from the ratio of signals (current/voltage) obtained by simultaneously measuring the voltage and current.

$$\left|Z\right|=\frac{V\left(f\right)}{I\left(f\right)}$$ 1

where *f* is the frequency. The potential used for the impedance measurement was 0 mV, the applied alternating current (AC) voltage was 100 mV, and the impedance |*Z*| was calculated from the response at the AC frequency (4 Hz to 8 MHz) at that time.

A protective hood was used to prevent the evaporation of water from the sample during the measurement.

## 3 Results and Discussion

### 3.1 Dynamic Viscoelastic Behavior during the Cooling Process of the Gelatin Solution

Figure 2 shows the change in the dynamic moduli of a 2.5% gelatin solution. The blue, green, and red plots are the cooling rate results of 1, 2, and 4 °C/min, respectively. The filled plots are the storage modulus *G*′, and the open plots are the loss modulus *G*″. At all of the cooling rates, *G*′ is zero for a while after the measurement begins. However, as the temperature decreased with time, the *G*′ was suddenly detected, and then it rapidly increased to 10^3^ Pa and approached a constant value. In addition, the point at which the elastic moduli rapidly increase shifts to the shorter time region with the increasing cooling rate. The apparent plateau modulus *G*′_p_, defined *G*′, approaches a constant value after a long time, decreasing from approximately 3000 Pa to approximately 1000 Pa with the increasing cooling rate (Figure 3). The reason why the *G*′_p_ changes with the cooling rate is reported to be due to, according to the viscosity measurement by Kawamura et al.,^19^ differences in the time required for collagen molecule orientation and, according to the electron microscope observation by Hinode and Kawamura,^28^ differences in the density of the network structure.

**Figure 2:**
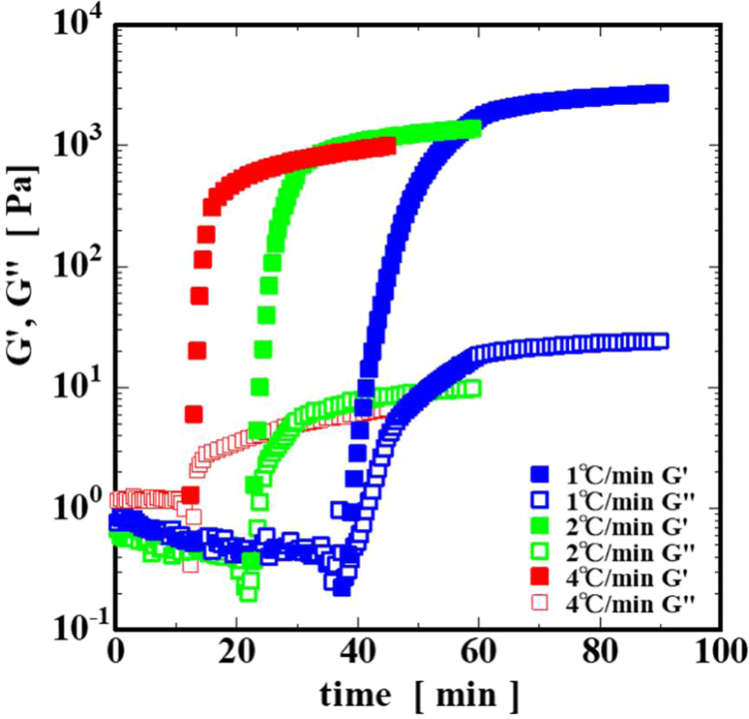
Temperature dependence of elastic moduli at cooling rates of 1, 2, and 4 °C/min.

**Figure 3:**
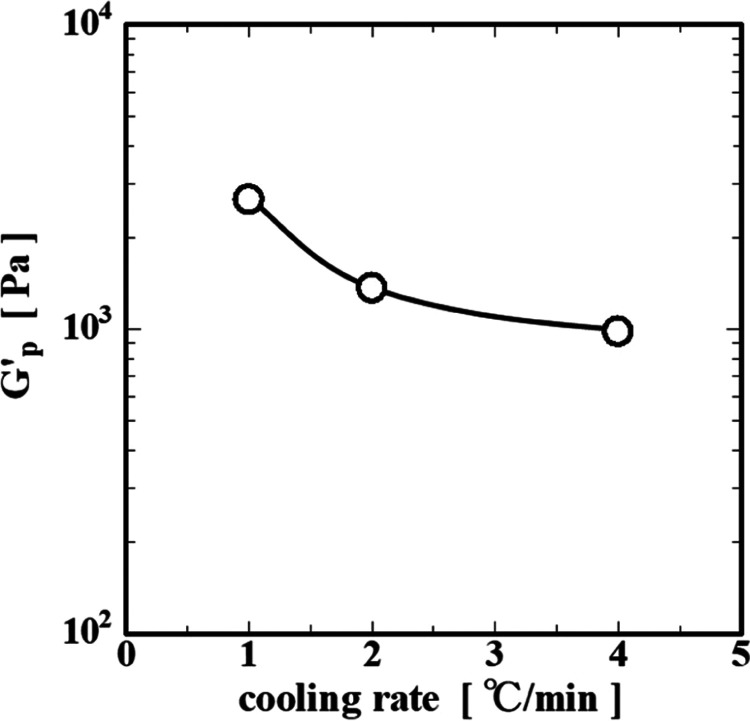
Dependence of plateau modulus *G*′_p_ on cooling rate.

On the other hand, *G*″ fluctuated around 10^–1^–10^0^ Pa for a while after starting the measurement, and after a certain time had passed, it suddenly increased to 10 Pa, as with *G*′, then gradually approached a constant value. Immediately after starting the measurement, the gelatin was a sol with a higher *G*″ than *G*′, but as the temperature dropped with time, *G*′ rapidly increased, and it transformed into a gel where *G*′ was higher than *G*″ via the intersection where G′ and *G*″ are equal.

The point where *G*′ and *G*″ coincide is considered to be the apparent sol–gel transition point (simply abbreviated as the gelation point), and the time and temperature at that point are plotted versus the cooling rate (Figure 4). As a result, when the cooling rate was increased, the gelation time decreased from 39 to 13 min (Figure 4a) and the gelation temperature decreased from 26 to 15 °C (Figure 4b). In the same way as the described behavior of the cooling rate dependence of *G*′_p_, it is postulated that when the cooling rate is slow, the molecules orient themselves and form a network structure, so the gelation occurs at a high temperature and in a short time, whereas with the rapid cooling rate, the molecules do not have time to orient themselves, so they form randomly a poor network structure, and in the meantime, the temperature of the solution rapidly decreases, resulting in a lower gelation temperature and a delay in time.^19^

**Figure 4:**
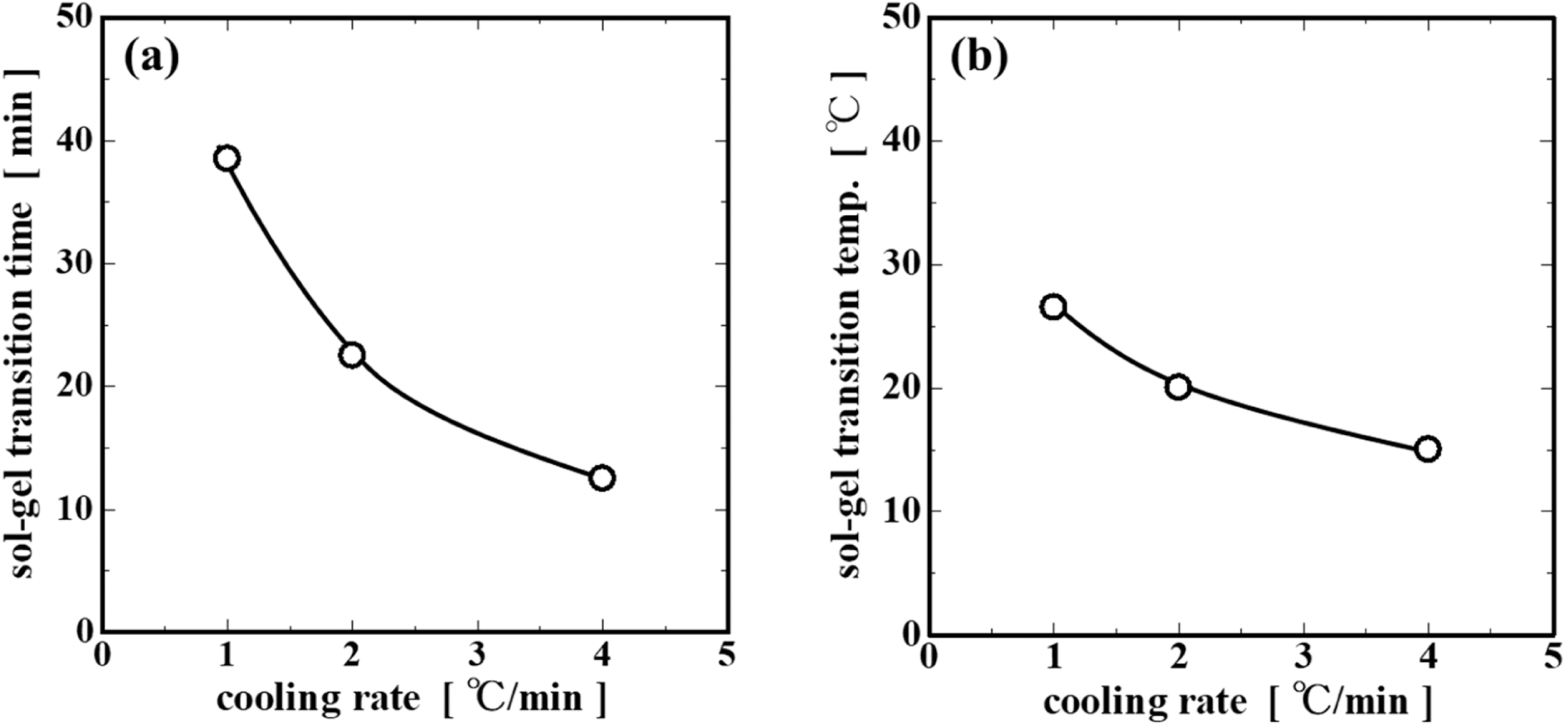
Dependence of the (a) sol–gel transition time and (b) sol–gel transition temperature on the cooling rate.

### 3.2 Impedance Behavior during the Cooling Process of the Gelatin Solution

The impedance of gelatin hydrogels increases with the gelatin concentration.^29,30^ This is because the gelatin molecular chains are chemically cross-linked, which makes the gelatin molecular chain network denser and finer, and the mobility of the electrolytes, such as sodium ions in a solvent, is suppressed. In other words, the electrical impedance of the gelatin gel depends on the interaction between the polymer chain network and electrolyte ions, and it is possible to capture changes in the internal network structure of the gel. However, their measurements were taken when the gelatin was a gel; therefore, the process from sol to gel could not be tracked. Therefore, we decided to evaluate the dynamic changes in their process using the rheo-impedance technique.

Figure 5 shows the Bode plots of the impedance |*Z*| at various temperatures during the cooling process of a 2.5% gelatin solution plotted versus the AC frequency *f*. Figure 5a–c shows the cooling rates of 1, 2, and 4 °C/min, respectively. Regardless of the cooling rate or the temperature, the slope of |*Z*| decreased at approximately −1 with the increasing AC frequency below 10^3^ Hz and remained almost constant in the intermediate frequency range of 10^3^ to 10^6^ Hz, but turned to a slow decrease again above the higher frequency of 10^5^ Hz. The impedance |*Z*| decreased in the low-frequency region below 10^3^ Hz due to the effect of the capacitance of the sample, and in general, as the frequency increased, the current flows more easily and the resistance (impedance) decreased. The decrease of the impedance |*Z*| in the high-frequency region above 10^6^ Hz is postulated to be due to either the instrument error or the effect of the instrument’s inherent resistance. Therefore, we focused on the impedance |*Z*| in the intermediate range of 10^4^ < *f* < 10^5^ Hz.

**Figure 5:**
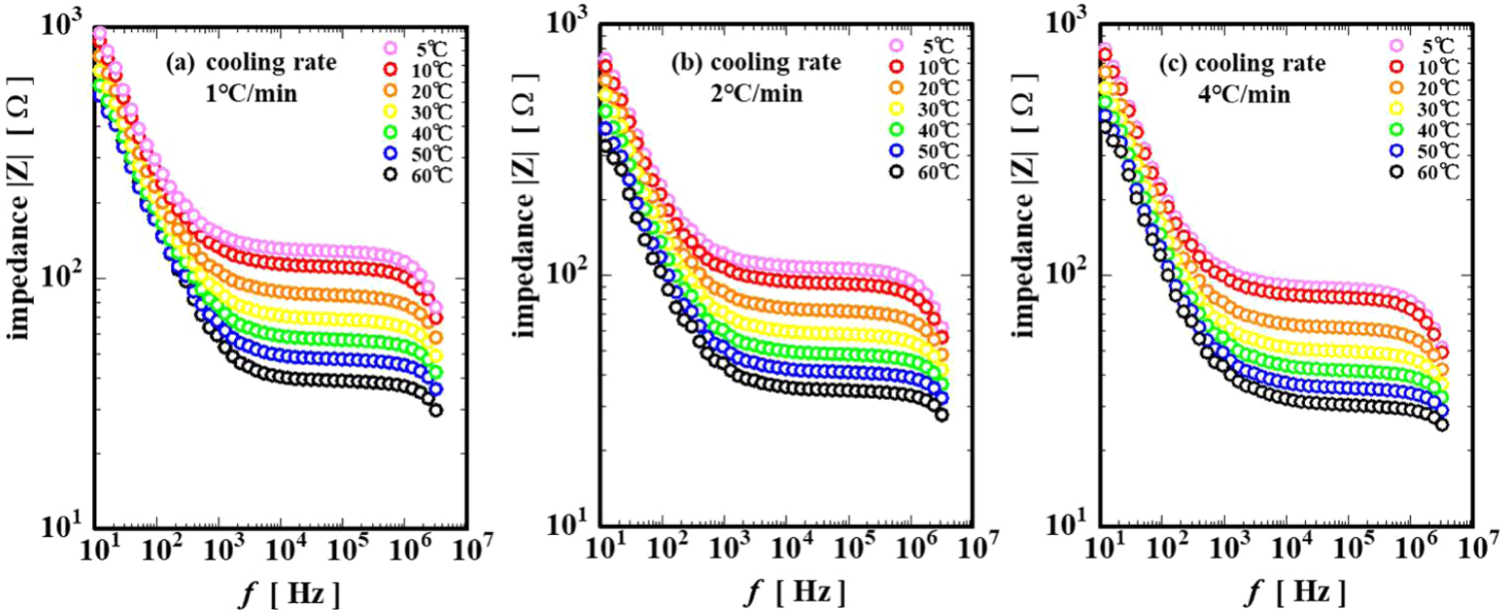
Changes in the Bode plots of gelatin solutions during the gelation process at cooling rates (a) 1 °C/min, (b) 2 °C/min, and (c) 4 °C/min.

Figure 6 shows the relationship between the impedance |*Z*| and temperature in the intermediate frequency range (3 × 10^4^ Hz). As shown in the figure, |*Z*| increases with decreasing temperature for all of the cooling rates. The impedance |*Z*| at the same temperature became lower with an increase in the cooling rate.

**Figure 6:**
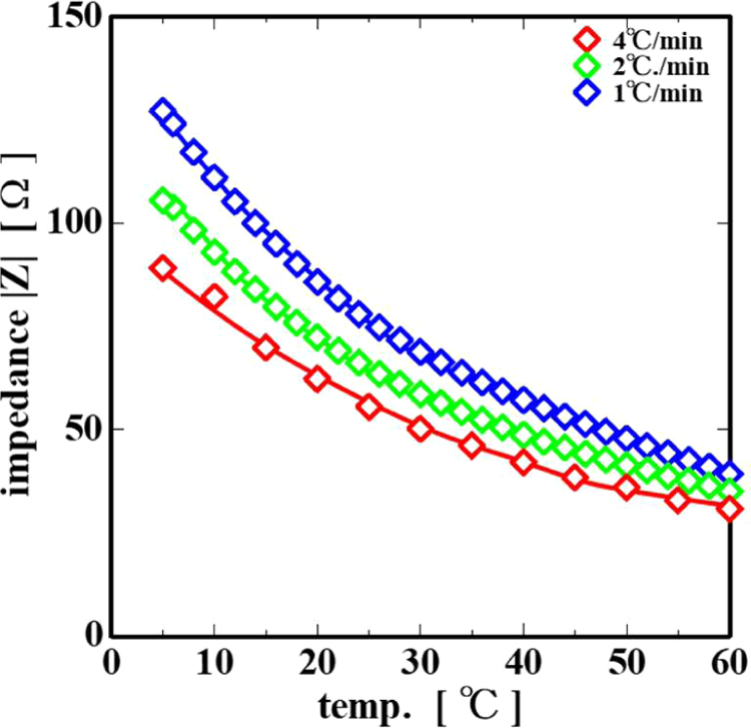
Temperature dependence of the impedance |*Z*| at 3 × 10^4^ Hz.

In general, the electrical resistance of a material changes with temperature. Usually, the resistance of metallic conductors increases with temperature, while that of semiconductors, electrolytic solutions, carbon, insulators, and other materials decreases. The electrical resistance of water is also affected by the temperature. This is because the dissociation constants of the hydrogen ion and hydroxide ion in water change with temperature. As the temperature decreases, the dissociation constant decreases and the electrical resistance (impedance |*Z*|) increases, as shown in Figure 7. In addition, a saline solution, which contains a large amount of electrolyte, has a low impedance and a slight temperature dependence. Furthermore, for both the purified water and saline solution, the impedance |*Z*| at the same temperature hardly changes even if the cooling rate is changed. However, in the gelatin solution shown in Figure 6, the impedance |*Z*| at the same temperature became lower with an increase in the cooling rate, indicating that the impedance depends on the cooling rate. The reason for this is discussed below.

**Figure 7:**
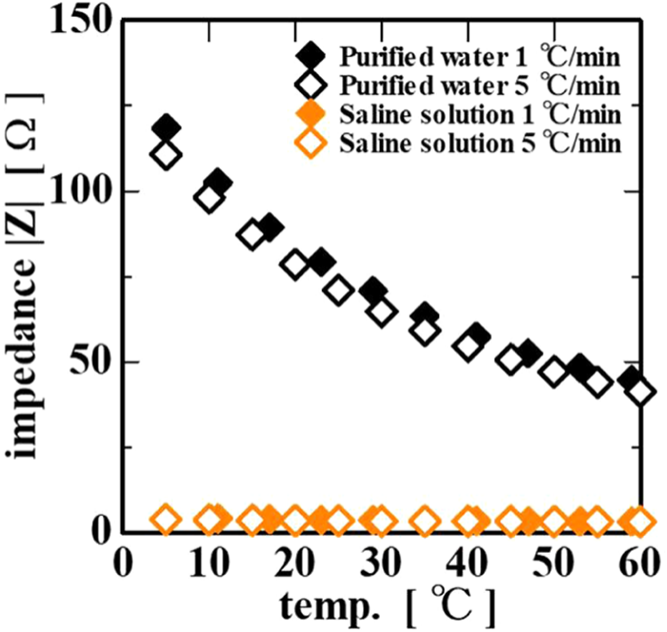
Temperature dependence of the impedance |*Z*| of purified water and saline solution at 3 × 10^4^ Hz.

### 3.3 Nyquist Plots and the Equivalent Circuit of the Gelatin Solution

Since the impedance |*Z*| is a complex number, it can be divided into a real component (*Z*′) and an imaginary component (*Z*″) as follows

2 $$\left|Z\right|=Z^{\prime }-jZ^{\prime \prime }$$

where *j* is the imaginary number, and *Z*′ and *Z*″ can be expressed as follows from the phase difference θ between AC Δ*V* and AC Δ*I*, and the |*Z*| value

3 $$Z^{\prime }=\left|Z\right|\cos\theta$$

4 $$Z^{\prime \prime }=\left|Z\right|\sin\theta$$

Based on the Bode plots of Figure 5a–c, the Nyquist plots^31^ were created by plotting *Z*′ on the *x*-axis and *Z*″ on the *y*-axis (Figure 6a–c). As a result, at all of the cooling rates, one semicircle appeared in the high-frequency region, and the size of the semicircle increased with decreasing temperature. In addition, the size of the semicircle tended to be larger with a slower cooling rate.

The electrochemical impedance measurement is a method for evaluating the electrical properties of the internal structure of a sample by simulating an equivalent circuit model based on the calculated electrical resistance (impedance |*Z*|). Based on the Nyquist plots obtained in Figure 8a–c, we decided to produce an equivalent circuit and consider the changes in the internal structure of the gelatin.

**Figure 8:**
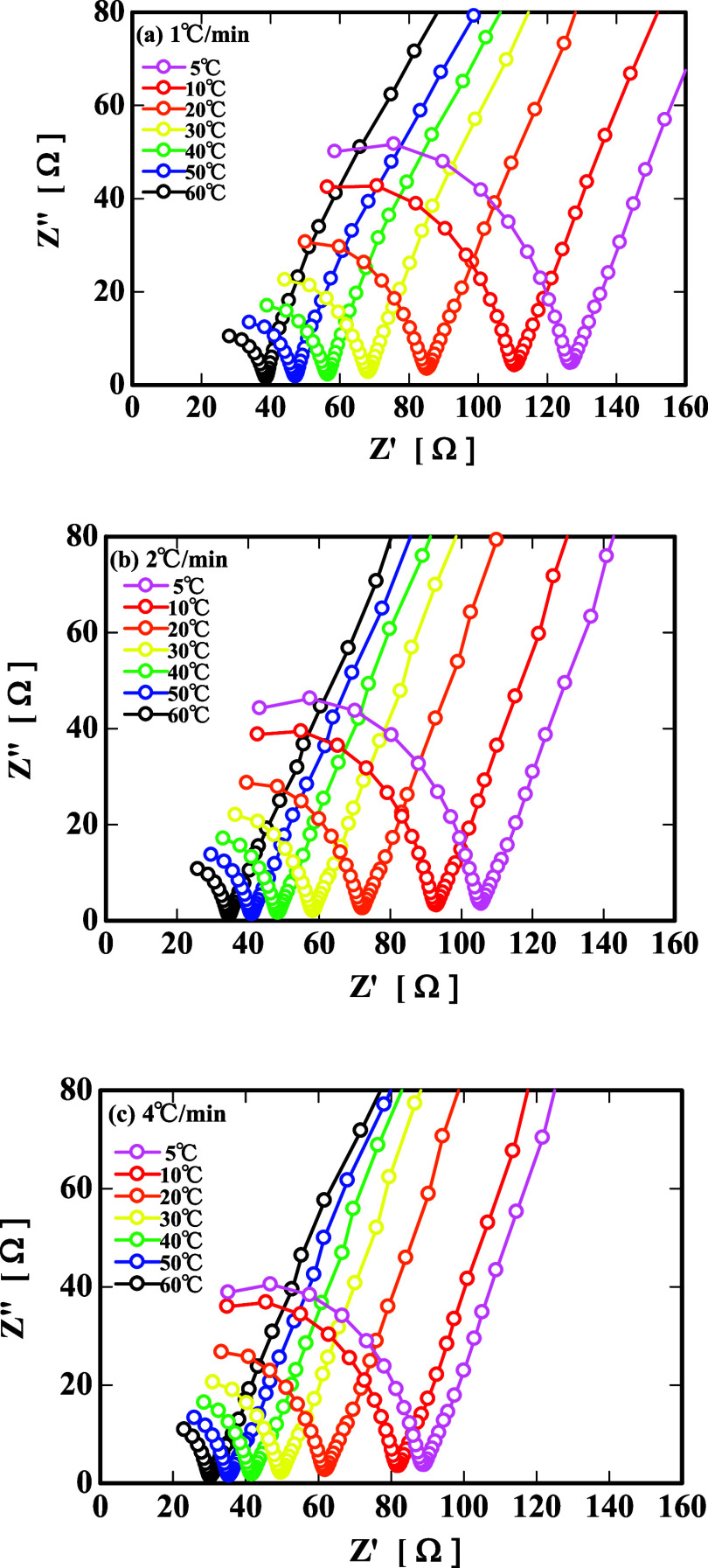
Temperature dependence of the Nyquist plots: (a) 1 °C/min, (b) 2 °C/min, and (c) 4 °C/min.

As one semicircle appeared in each of the Nyquist plots in Figure 8a–c, we considered that the equivalent circuit of the gelatin could be expressed, as shown in Figure 9.

**Figure 9:**
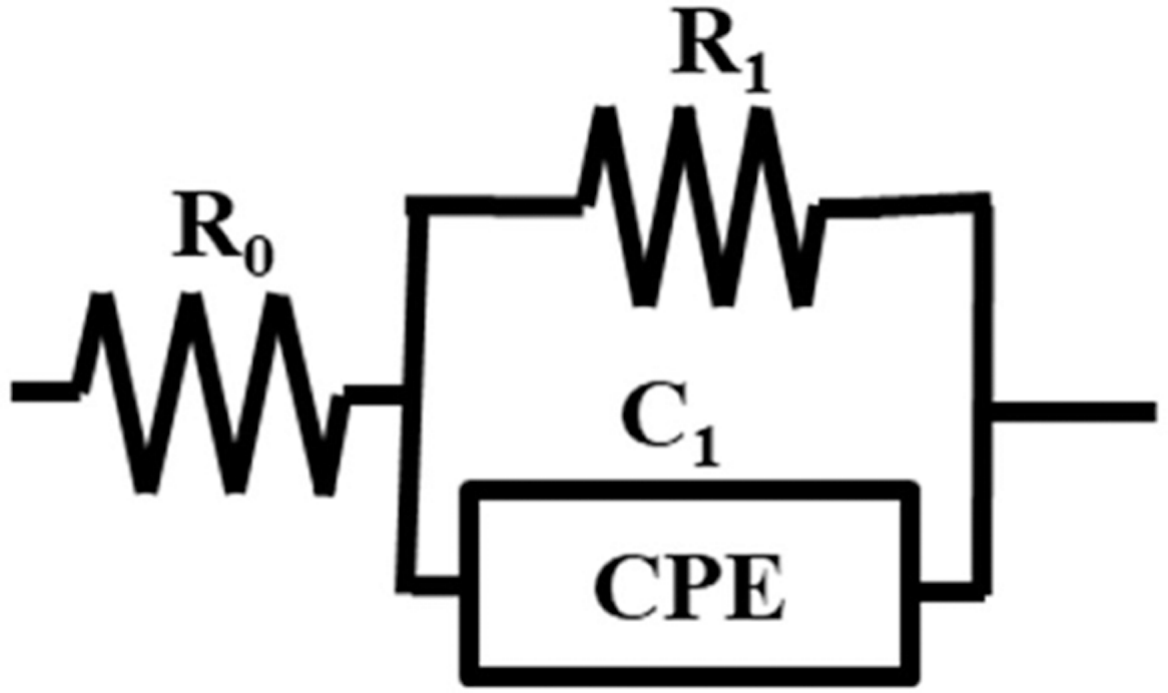
Equivalent circuit of the gelatin solution.

In general, the equivalent circuit can be represented by a combination of the resistance *R* and capacitance *C*; however, we used a constant-phase element, CPE, instead of the capacitance, *C*, because the semicircle is collapsed in the real axis direction. In other words, we theorized that it could be expressed as a model with a parallel circuit of resistance, *R*_1_, and a constant-phase element, CPE, connected in series with resistance, *R*_0_. *R*_0_ represents the resistance of the solvent, and since it is assumed that electrolytes, such as salts contained in gelatin, are dissolved in the solvent, its value is almost zero. In addition, the parallel connection model of *R*_1_ and CPE is considered to be related to the internal resistance of the gelatin. The equivalent circuit shown in Figure 9 can be expressed by the theoretical formula shown by eq 5.^32,33^

$$\left|Z\right|=R_{0}+\frac{R_{1}}{1+{\left(j\omega R_{1}C_{1}\right)}^{p}}$$ 5

The impedance spectra were analyzed using Scribner’s ZView^34,35^ with complex nonlinear least-squares (CNLS) curve fitting to calculate the circuit parameters. The calculated *R*_1_ was assumed to be the resistance inside the sample of the system. The maximum iteration was set to 100 so that the standard error of *R*_1_ was less than 1%.

Figure 10 shows the change in the gelation process internal resistance *R*_1_ calculated using ZView. *G*′ and *G*″ values shown in Figure 2 are also plotted in Figure 10. As can be seen from Figure 10, at all the cooling rates, *R*_1_ can be approximated by an exponential function as shown in eq 6 up to the point where the gelation is completed (the elastic moduli approach a certain value), that is, from the sol to near the sol–gel transition.

6 $$R_{1}=ae^{\mathrm{bt}}$$

where *a* and *b* (power-law index coefficients) are constants and *t* is the time. The power index coefficient b in Formula [Disp-formula eq6] linearly increased with the cooling rate; therefore, we found that the internal resistance of the system rapidly increased over a short period of time with the cooling rate (Figure 11). In addition, *R*_1_ near the end of gelation showed a lower value with an increase in cooling rate.

**Figure 10:**
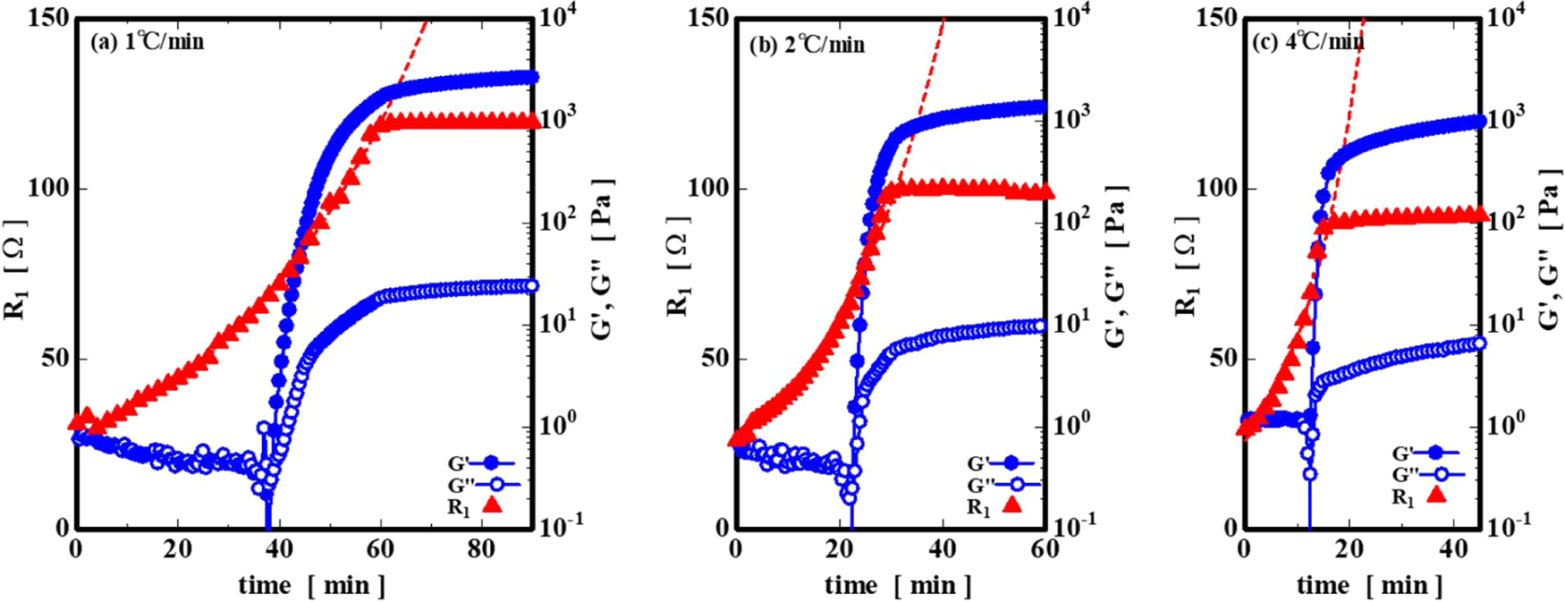
Temperature dependence of the internal resistance *R*_1_ at the cooling rate of (a) 1 °C/min, (b) 2 °C/min, and (c) 4 °C/min (The elastic moduli in Figure 2 are also overlaid).

**Figure 11:**
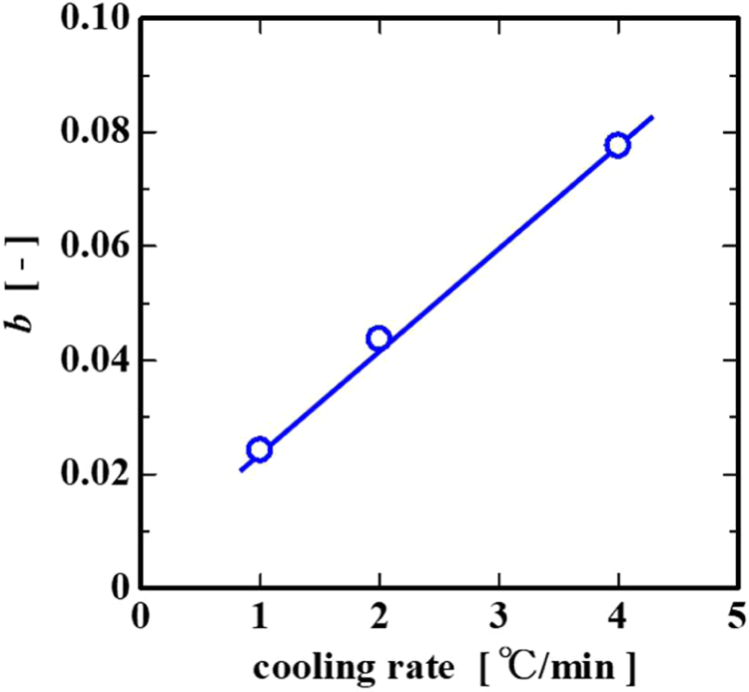
Relationship between the power-law index coefficient *b* and the cooling rate.

The temperature dependence of the internal resistance, *R*_1_, showed the same tendency as the behavior of the impedance |Z| shown in Figure 6, in that *R*_1_ tended to decrease with the increasing cooling rate even at the same temperature (Figure 12). This is considered to be because the electrochemical properties of the gelatin are not simply dependent on the temperature but are also related to structural factors such as the internal structure of the system. In other words, we assumed that *R*_1_, which exponentially increases from the sol to near the sol–gel transition, may indicate the resistance of the electrolytes and ions moving within the cross-linked collagen molecules. The gelatin is usually a sol at high temperatures and maintains a flexible random coil structure. When the gelatin solution is cooled, the collagen molecules form a partial helix structure. When these helix structures join to form a network, they lose their liquidity and become a gel. The simple model of the gelation of the gelatin described above is shown in Figure 13.

**Figure 12:**
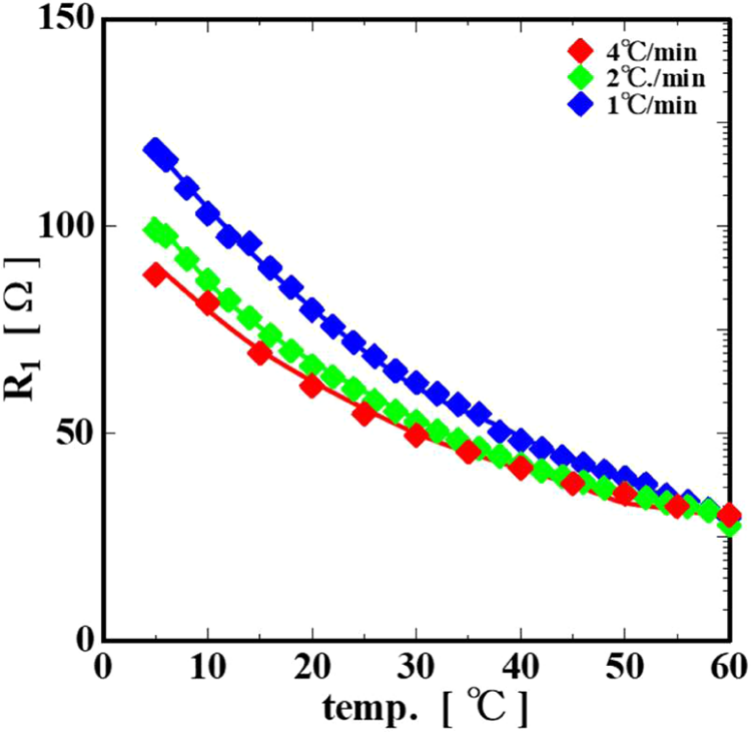
Temperature dependence of internal resistance *R*_1_.

**Figure 13:**
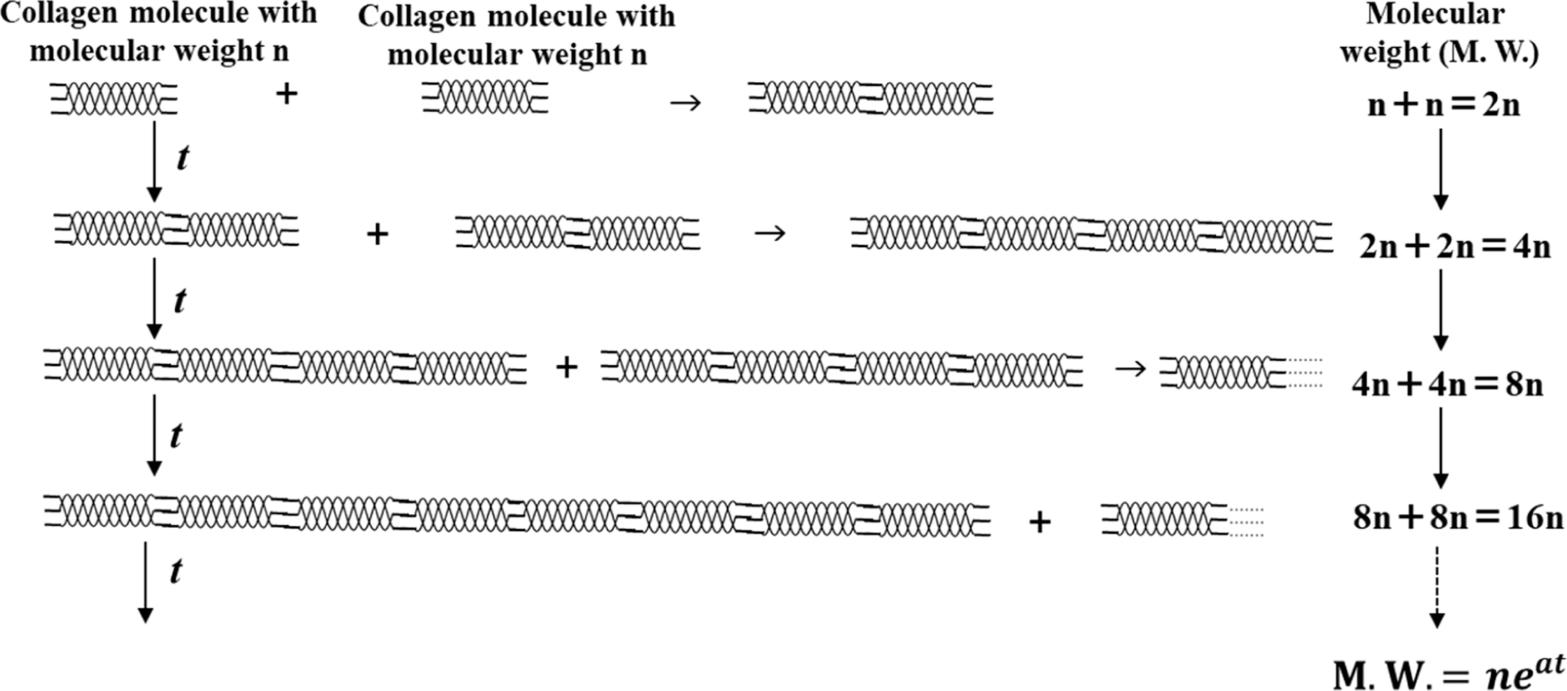
Schematic diagram of the change in the molecular weight associated with the cross-linking reaction of the collagen molecules in the gelatin.

First, if collagen molecules with a molecular weight of *n* are cross-linked, then the molecular weight of the resulting dimer collagen will be 2*n*. Next, if collagen molecules with a molecular weight of 2*n* cross-link with each other, then the molecular weight will be 4*n*. Furthermore, if the same cross-linking continues to progress, the molecular weight of the resulting collagen molecules will exponentially increase over time. The cross-linking reaction of actual collagen molecules cannot be expressed by a simple model such as the one described above, but we surmised that the exponentially increasing internal resistance, *R*_1_, corresponds to the increasing molecular weight of the collagen molecules as the cross-linking progresses.

Furthermore, as shown in Figure 14, we found that there is a proportional relationship between *G*′_p_ and *R*_1_ in the region where the gelation is converging. *G*′_p_ and *R*_1_ are thought to be the characteristic values related to the cross-linking density of the gelatin gel and the molecular weight of the collagen molecule, respectively. Therefore, although it may be indirect, we can infer that there is a relationship between the increase in the molecular weight of collagen and the formation of a dense network structure.

**Figure 14:**
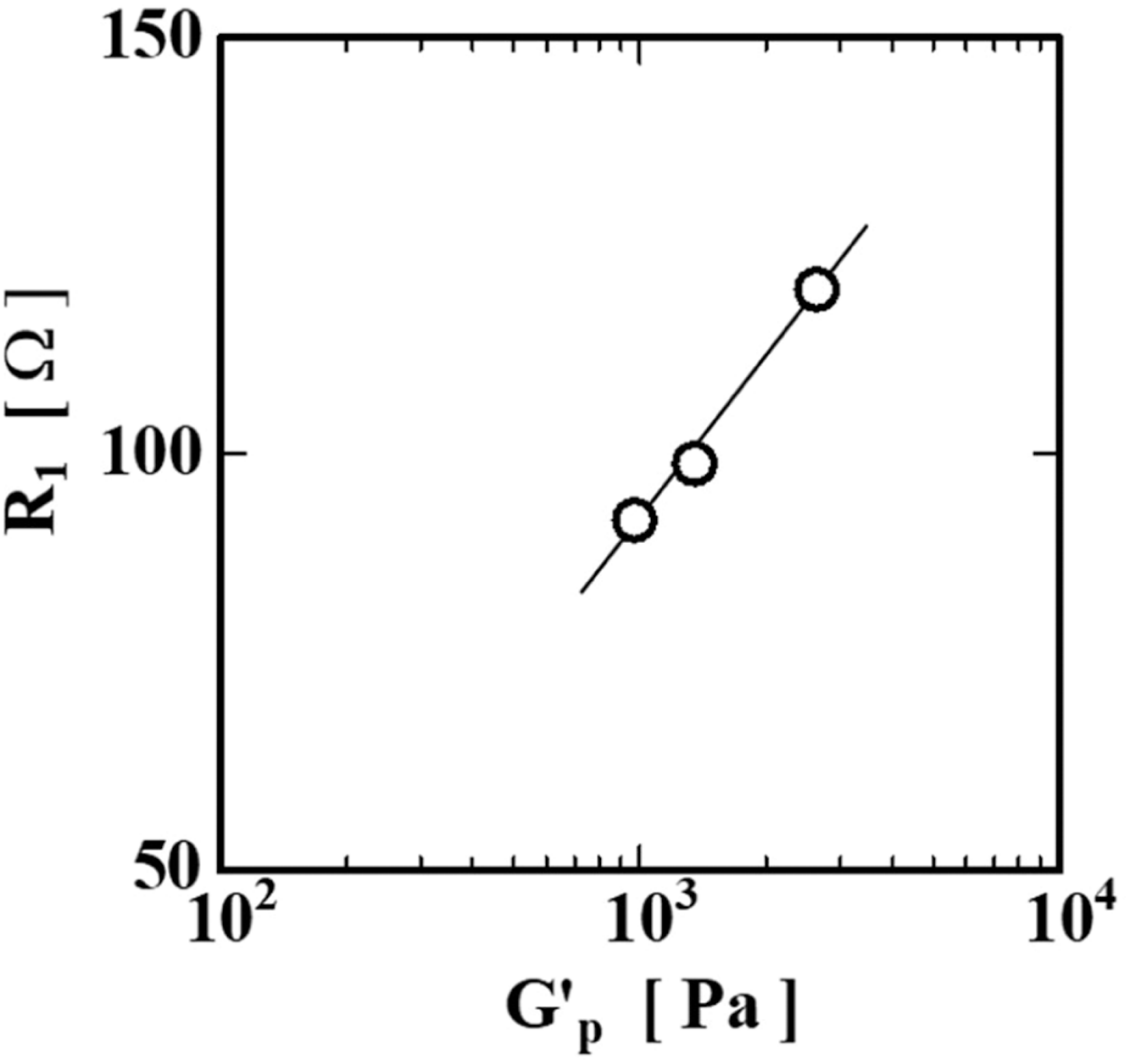
Relationship between internal resistance *R*_1_ and plateau modulus *G*′_p_.

Summarizing these, we inferred that the increase in the internal resistance, *R*_1_, of the gelatin solution was related to the cross-linking growth (molecular weight) of the collagen molecules, because *R*_1_, calculated from the impedance measurement, exponentially increased from the sol to near the sol–gel transition. In addition, *R*_1_ is also proportional to the plateau modulus *G*′_p_, which is related to the density of the collagen molecule network, and these results also support the above-mentioned inference.

## 4 Conclusions

We evaluated the dynamic and real-time changes in the internal structure of a gelatin solution from the sol to gelation process using the rheo-impedance technique.

The point at which the storage modulus, *G*′, and loss modulus, *G*″, of the gelatin rapidly increased shifted to a lower temperature and a shorter time with the increasing cooling rate. In addition, the *G*′ value at the point where the gelation converged was higher for the slower cooling rates. Since the gelatin network density is more densely formed for the slower cooling rates, *G*′ can be said to be a characteristic value related to the network density.

In the gelation process, especially in the process from the sol to near the sol–gel transition point, which was difficult to quantitatively evaluate in viscoelasticity measurements, we found that the impedance |*Z*| increased in real time with decreasing temperature. The impedance |*Z*| during the gelation process increased with decreasing temperature. The resistance value, *R*_1_, which is thought to be due to the internal structure of the gelatin, exponentially increases from the sol to near the sol–gel transition. Based on these results, it seems that *R*_1_ indicated the resistance of electrolytes and ions moving inside the cross-linked collagen molecules. In addition, since the apparent plateau modulus, *G*′_p_, which is related to the network structure (molecular weight within the cross-linking structure) of the collagen molecules, is also in a proportional relationship with *R*_1_, we concluded that *R*_1_ corresponds to the increase of molecular weight accompanying the progress of the cross-linking.

As already mentioned, the simultaneous measurement of the rheological and electrochemical properties such as the rheo-impedance measurement makes it possible to capture in real time changes in the physical properties during the gelation process of a gelatin solution, which was previously difficult, and it can also be said to be a very effective method for examining the dynamic changes in internal structure.
